# Extracellular Self-DNA Effects on Yeast Cell Cycle and Transcriptome during Batch Growth

**DOI:** 10.3390/biom14060663

**Published:** 2024-06-06

**Authors:** Emanuela Palomba, Maria Luisa Chiusano, Francesco Monticolo, Maria Chiara Langella, Massimo Sanchez, Valentina Tirelli, Elisabetta de Alteriis, Marco Iannaccone, Pasquale Termolino, Rosanna Capparelli, Fabrizio Carteni, Guido Incerti, Stefano Mazzoleni

**Affiliations:** 1 Institute of Biosciences and Bioresources CNR, Via Università 133, 80055 Portici, Italy; emanuela.palomba@ibbr.cnr.it (E.P.); pasquale.termolino@ibbr.cnr.it (P.T.); 2 Department of Agricultural Sciences, University of Naples “Federico II”, Via Università 100, 80055 Portici, Italy; chiusano@unina.it (M.L.C.); francesco.monticolo@unina.it (F.M.); mariac.langella@studenti.unina.it (M.C.L.); rosanna.capparelli@unina.it (R.C.); fabrizio.carteni@unina.it (F.C.); 3 Cutaneous Biology Research Center, Massachusetts General Hospital, Boston, MA 02129, USA; 4 Istituto Superiore di Sanità (ISS) Core Facilities, Viale Regina Elena 299, 00161 Rome, Italy; massimo.sanchez@iss.it (M.S.); valentina.tirelli@iss.it (V.T.); 5 Department of Biology, University of Naples “Federico II”, Via Cinthia 26, 80126 Naples, Italy; dealteri@unina.it; 6 Laboratory of Bioproducts and Bioprocesses ENEA, Piazzale Enrico Fermi 1, 80055 Portici, Italy; marco.iannaccone@enea.it; 7 Department of Agricultural, Food, Environmental and Animal Sciences, University of Udine, Via delle Scienze 206, 33100 Udine, Italy; guido.incerti@uniud.it

**Keywords:** self-DNA inhibition, *Saccharomyces cerevisiae*, RNA-seq, cell growth

## Abstract

The cell cycle and the transcriptome dynamics of yeast exposed to extracellular self-DNA during an aerobic batch culture on glucose have been investigated using cytofluorimetric and RNA-seq analyses. In parallel, the same study was conducted on yeast cells growing in the presence of (heterologous) nonself-DNA. The self-DNA treatment determined a reduction in the growth rate and a major elongation of the diauxic lag phase, as well as a significant delay in the achievement of the stationary phase. This was associated with significant changes in the cell cycle dynamics, with slower exit from the G0 phase, followed by an increased level of cell percentage in the S phase, during the cultivation. Comparatively, the exposure to heterologous DNA did not affect the growth curve and the cell cycle dynamics. The transcriptomic analysis showed that self-DNA exposure produced a generalized downregulation of transmembrane transport and an upregulation of genes associated with sulfur compounds and the pentose phosphate pathway. Instead, in the case of the nonself treatment, a clear response to nutrient deprivation was detected. Overall, the presented findings represent further insights into the complex functional mechanisms of self-DNA inhibition.

## 1. Introduction

In microbial communities, extracellular DNA (exDNA) is widely found [1,2,3] and may originate from cell lysis or can be actively released by living cells [4]. Many different cultivated bacterial species, such as *Bacillus* [5], *Pseudomonas* [6], and *Neisseria* [7], as well as different environmental isolates [8] are known to secrete exDNA in their external *milieu*.

In many cases, such actively released exDNA accumulates in the exopolymeric matrix of bacterial biofilms [9], where it contributes to the structural organization of the matrix and protection from host defense responses [4]. In fungi, exDNA has also been reported to be a component of the extracellular matrix of *Candida albicans* [10] and *Aspergillus fumigatus* [11] biofilms.

A species-specific inhibitory effect of extracellular self-DNA was reported in plants and several other organisms [12,13], whereas no inhibitory effect was observed in all species when treated with heterologous exDNA. In particular, such an inhibitory effect was revealed in both marine and freshwater microalgae, with a typical palmelloid resting state in *Chlamydomonas reinharditi* and the formation of aggregates and biofilm [14].

Recently, for the first time, the active release and accumulation of extracellular DNA in the medium of fed-batch cultures of the yeast *Saccharomyces cerevisiae* has been demonstrated [15]. In this work, an inhibitory action of such self exDNA was reported on yeast proliferation, reducing the final cell density of the population, notwithstanding the maintenance of nutrient feeding in fed-batch runs. The exDNA recovered from the medium was found to be single stranded and corresponded only to a fraction of the entire yeast genome, thus showing that it did not derive from cell lysis. Interestingly, high throughput sequencing revealed that the exDNA sequences were different in relation to either the fermentative or respiratory status of the yeast cells during cultivation. Flow cytometry analysis of the yeast population cell cycle along the time course of the fed-batch run showed an unexpected percentage of cells arresting in the S phase [15].

In the same work, it was also reported that the inhibitory effect of exDNA on yeast growth was maintained in the exhausted medium and that it could be removed via the adsorption on hydroxyapatite (HAP). This HAP-based method, exploiting the interaction between positively charged calcium ions on HAP and the negatively charged phosphate backbone of the nucleic acids, has been routinely used, since the 1960s [16], for the fractionation and recovery of DNA from different origins [17,18].

Here, we further investigated the effects of an exhausted medium, containing specific DNA fragments secreted in fermentative conditions, on cell cycle and transcriptome dynamics during an aerated batch culture on glucose. As widely described [19,20], *S. cerevisiae* growth in a typical batch system is characterized by an initial exponential fermentative phase on glucose, leading to ethanol production. This is followed by a so-called diauxic lag phase, in which a metabolic switch activates the respiratory machinery, thus starting a second oxidative exponential phase using the accumulated ethanol as a substrate. Finally, with the end of nutrient availability in the vessel, a plateau, leading to a stationary growth phase, is observed [21].

The aim of this study was to investigate the cell cycle and transcriptome dynamics of yeast exposed to the effect of self- or (heterologous) nonself-exDNA in the growth medium, also addressing the following specific objectives: (i) to analyze the inhibitory effect of self-DNA present in fermentative supernatant during the different batch phases; (ii) to confirm that the removal of such self-DNA from the growth medium eliminates the inhibitory effect; (iii) to assess the lack of inhibitory effect resulting from the addition of heterologous DNA to the growth medium, also at high concentration levels.

## 2. Materials and Methods

### 2.1. Strain and Medium

The strain selected for the study was *S. cerevisiae* CEN.PK2-1C (*MATa ura3*-*52 his3*-*D1 leu2*-*3,112 trp1*-*289 MAL2*-*8c SUC2*), purchased from the EUROSCARF collection (www.uni-frankfurt.de/fb15/mikro/euroscarf (accessed on 6 October 2014)).

The medium used to culture the yeast strain was the same one used in previous fed-batch experiments [15]. It was prepared according to [22] with 2% *w*/*v* glucose, vitamins, and trace elements and was supplemented with 10 g L^−1^ of casamino acids (BD Bacto TM Casamino Acids, BectonDickinson & Co., Sparks, MD, USA). Uracil, histidine, leucin, and tryptophan were added to fully cover the yeast strain request [23].

### 2.2. Culture Conditions

Aerobic batch cultures were performed in 250 mL flasks containing 80 mL of the culture medium described above. Flasks were inoculated with an adequate aliquot of a pre-culture of the CEN.PK2-1C strain in the exponential phase, to give an initial optical density at 590 nm (O.D._590_) of 0.15, and were then incubated up to 144 h at 28 °C and 200 rpm.

In addition to the control (C), three different treatments were considered, supplementing the culture medium with (i) the exhausted medium (I) recovered from a previous exponential fed-batch culture containing fragments of exDNA secreted during fermentative metabolism [15]; (ii) the same exhausted medium after removal of exDNA using HAP (R), and (iii) heterologous DNA at a concentration of 350 µg mL^−1^ (H).

As heterologous DNA (nonself-DNA), a commercial fish sperm ssDNA (Roche Diagnostics, Almere, The Netherlands), provided as a pure, sonicated, denatured, single-stranded DNA fragment mixture (chain length 120 to 3000 nucleotides), was used.

In the case of the exhausted medium (I) and of the same medium after DNA removal (R), they were added to the substrate up to a final concentration of 75% *v*/*v*.

During incubation, samples were withdrawn at different time intervals (2, 6, 11, 24, 48, 72, 96, and 144 h) from each of the four cultures (named C, I, R, H), to monitor yeast growth by O.D._590_ (2.30 O.D._590_ per g biomass L^−1^). Three biological replicates for each treatment were performed.

### 2.3. Preparation of the Media for the I and R Cultures

The exhausted medium collected at the end of a previous fed-batch culture, with exponential glucose feeding and containing exDNA fragments secreted in fermentative conditions [15], was centrifuged (3000× *g*, 5 min) and then filtered (Stericup^®^ Millipore Express^®^, Burlington, MA, USA, 0.22 μm diameter). Ethanol in the exhausted medium was reduced to 0.03% *v*/*v*, following vacuum distillation at 37 °C using a Buchi R-215 (Büchi Labortechnik AG, Flawil, Switzerland) rotary evaporator. The resulting I medium was used for the growth experiments.

To obtain the R medium, an aliquot (250 mL) of the filtered exhausted medium I, was loaded on a HAP to remove exDNA. The medium was iteratively loaded on a HAP (Hydroxyapatite DNA grade: Bio-Gel HTP) column, which was previously adapted in 0.005 M phosphate buffer pH 6.8, pre-heated at 60 °C. Then, possible residual exDNA still present in the medium was determined via fluorimeter Qubit™ 3.0 using a Qubit dsDNA HS Assay Kit and Qubit ssDNA Assay Kit, (Life Technology, Carlsbad, CA, USA), resulting to be lower than the detection limits.

### 2.4. Cytofluorimetry

The cell cycle dynamics were studied during the aerobic batch cultures C, I, R, and H, by assessing the DNA content in the yeast cells using cytofluorimetric analysis. To this end, yeast cell samples, collected from the four cultures at different time points (2, 6, 11, 24, 48, 72, 96, and 144 h), were centrifuged (3000× *g*, 5 min) to obtain cell pellets. Then, cells were resuspended and fixed in 75% *v*/*v* ethanol, added dropwise under continuous vortexing to avoid cell agglomeration.

Fixed cells were centrifuged, treated with 1 mg mL^−1^ DNase-free RNAse A (Sigma-Aldrich, St. Louis, MO, USA), and stained using SYTOX Green (1 μM, Invitrogen™, Waltham, MA, USA, λex 504 nm/λem 523 nm) as a DNA binding dye [24]. Cells were acquired using a Gallios Flow cytometer, equipped with three lasers (405 nm, 488 nm, and 633 nm, Beckman Coulter, Milan, Italy), and data were analyzed using Kaluza Analysis Software v. 2.1 (Beckman Coulter).

In parallel, from a batch culture set up at 28 °C with the same culture medium, yeast cells were collected at O.D._590_ = 0.6 (exponential cells) and after seven days (starved cells), to be used as reference samples, and processed for flow cytometric analysis, as reported above.

### 2.5. RNA Sampling, Extraction, and Sequencing

Yeast cells were collected from the four cultures (C, I, R, and H) at different time points (2, 6, 11, 24, 48, 72, 96, and 144 h). Following harvesting, 1 × 10^8^ cells were centrifuged at 5000 rpm for 5 min and washed 3 times with PBS (cat P3813, Sigma-Aldrich). The ells were resuspended directly in Trizol reagent (Cat num: 15596026, Thermo Fisher Scientific, St. Louis, MO, USA) and glass spherical beads (Ø 425–600 μm, Sigma-Aldrich) were added. Yeast cells were mechanically lysed by several cycles (7 cycles) of mixing (200 rpm) for 1 min alternate to ice incubation for 1 min. Following cell lysis, total RNA was obtained following the manufacturing instruction’s (Trizol manual instructions). Total RNA was resuspended in nuclease-free water (Cat num: 4387936, Thermo Fisher Scientific) and the RNA yield was monitored using a NanoDrop ND 100 spectrometer (NanoDrop Technologies, Wilmington, DE, USA). RNA integrity was verified using the 2100 Bioanalyzer (Agilent Technologies, Santa Clara, CA, USA) and only samples with a RIN ≥ 9 were used for further analysis. RNA samples were sent to a service provider for the RNA-seq analysis on Illumina Hiseq2500, Novogene, using single read sequencing 1 × 15 M.

### 2.6. Bioinformatics

The raw data quality was assessed using the FASTQC tool version 0.12.1 [25]. The cleaning of the remaining adaptors and the trimming of the low-quality bases at 5′ and 3′ positions of the reads were performed using Trimmomatic [26] (settings: minimum quality per base at 30 phread score; minimum length of the read after cleaning at 100 bp). The cleaned reads were mapped onto the *Saccharomyces cerevisiae* genome (including mitochondrion, viruses, and plasmids (Strain: S288C, version R64)) using the STAR aligner [27]. The maximum number of mismatches allowed was set to 10.

The mapped reads were counted per exon on yeast genes using FeatureCounts (release 1.6.3) [28] with the overlapping option and other parameters as default values and considering the gene annotation version 3.1.

The consistency of the replicates was tested via multivariate ordination and classification of the mapping results, based on Principal Component Analysis (PCA) and numerical clustering, respectively. The PCA results were presented by plotting the replicates of each combination of treatment and timing in the bidimensional space, as defined by the first two Principal Components, representing the largest fraction of the total variance of the input dataset. Numerical clustering was conducted using the Euclidean distance as the between-groups metric (calculated on standardized data) and Ward’s method as the aggregation rule. The results were presented as a dendrogram of the replicates.

Differential expression analysis was performed by calculating fold changes using EdgeR [29]. Only genes with |log2Fold change| ≥ 1 and FDR < 0.05 were considered as significant Differentially Expressed Genes (DEGs).

Log2 of the median of TMM-normalized gene expression calculated using EdgeR (log2TMM) was also defined for each of the 6600 yeast protein-coding genes.

Gene Ontology (GO) enrichment analysis was performed using gProfiler (https://biit.cs.ut.ee/gprofiler/gost (first accessed on 5 January 2024)) (Ensembl 102, Ensembl Genomes 49) [30]. Only GOs with FDR < 0.05 were considered as significant.

Pathway descriptions for yeast were obtained from the BioCyc Genome Database Collection (https://yeast.biocyc.org (accessed on 5 February 2024)) version 28.0 [31].

## 3. Results

### 3.1. Aerobic Batch Cultures and Cytofluorimetric Cell Cycle Analysis

**Figure 1:**
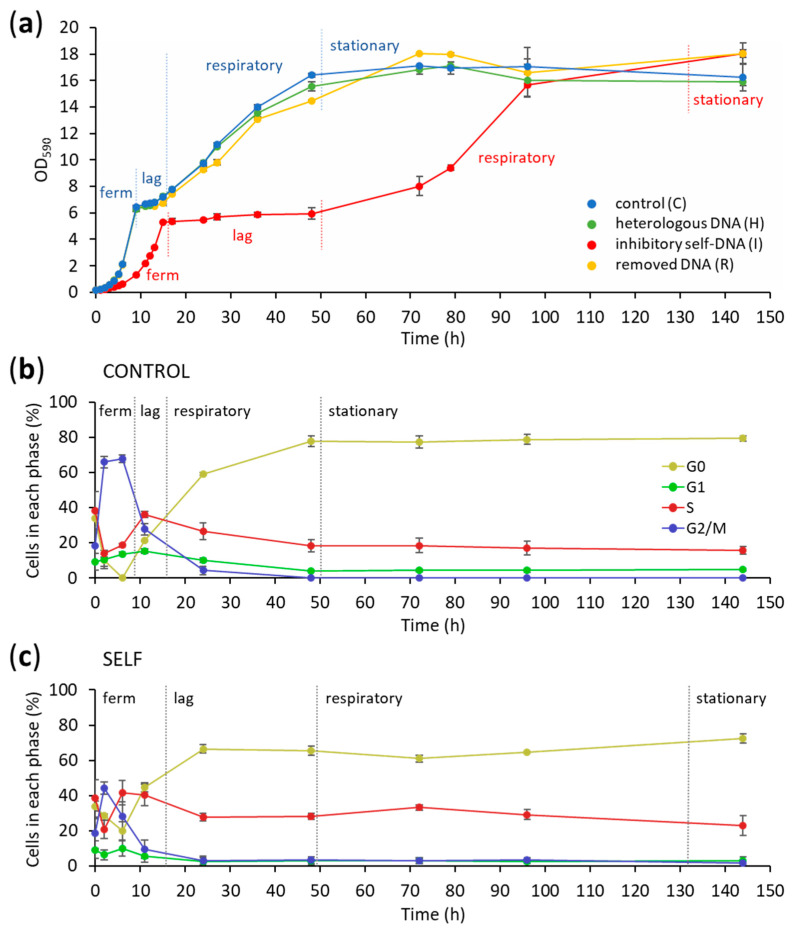
Growth and cell cycle profiles of yeast populations in batch cultures under different conditions (C, H, I, and R). (**a**) Growth curves of the four culture conditions. Vertical dashed lines indicate the separations between the main growth phases: glucose fermentation, diauxic lag phase, respiratory phase, and, finally, stationary phase. (**b**) Dynamic trends of the control yeast population in the different cell cycle phases (G0, G1, S, and G2/M). (**c**) Dynamic trends of the yeast population grown with the exhausted medium containing extracellular self-DNA in the different cell cycle phases (G0, G1, S, and G2/M). Data are averages of three biological replicates ± SD.

The time course of yeast growth during 144 h incubation in four different cultures with (i) control medium (C), (ii) medium containing yeast inhibitory self-DNA (I), (iv) medium from which DNA was removed (R), and (iv) heterologous DNA (H) are reported in Figure 1a.

As expected, yeast growth in the control culture is typically diauxic, with a first fermentative phase on glucose (lasting 8 h), followed by a lag phase (5 h), and then a second respiratory exponential phase on ethanol (lasting 35 h), before the achievement of stationarity due to the exhaustion of nutrients. The same growth trend was observed in the case of R and H cultures, whereas major differences in the growth curve occurred in the presence of the exhausted medium (I) (Figure 1a).

In this treatment, the growth curve appeared strongly delayed, with longer fermentative and respiratory phases (15 and 45 h, respectively, instead of 8 and 35 h reported for the control), with a diauxic lag phase lasting about 35 h (instead of the 5 h observed in the control and other treatments). In detail, in the I culture, the specific growth rate on glucose was strongly reduced (0.38 vs. 0.50 h^−1^) in the first fermentative phase, and a further reduction (0.03 vs. 0.04 h^−1^) was still present in the second respiratory phase on ethanol. However, despite such differences, after the second exponential phase, the yeast population in the I culture finally achieved a similar plateau level as in C, R, and H cultures (Figure 1a).

Regarding the cytofluorimetric analysis, the overall trends of the relative distribution of different cell cycle stages (G1, S, G2/M, and G0) during 144 h of cultivation are reported in Figure 1b,c for the C and I cultures, respectively.

Starting from the inoculum, the control culture showed a significant increase in the fraction of cell population in the G2/M phase, reaching almost 70% until 8 h, with cells in active division while fermenting glucose. This faction then decreased when approaching the lag phase and then continued to decrease during the respiratory growth phase, finally reaching a 0 value at stationarity at 50 h (Figure 1b). In parallel, the fraction of cells in the S phase, after an early decrease, reached a maximum of almost 40% at the beginning of the lag phase, then progressively decreased to 20%, while G0 showed an opposite increasing trend, stabilizing at a value above 70% at growth plateau at 50 h (Figure 1b).

Instead, in the case of the I culture, such an increase in the G2/M fraction was limited (about 45%), with an earlier decrease to less than 10% before the onset of the delayed lag phase around 10 h (Figure 1c). During the respiratory growth phase and until the end of the experiment, a constant low level of about 5% was maintained, with a small reduction at 144 h. The fraction of cells in the S phase reached its maximum at about 40% during the fermentative exponential growth phase, followed by a reduction to a stable value of 30% during the elongated lag phase until 50 h. During the second respiratory exponential growth phase, after a slight increase at 70 h, the fraction of cells in the S phase progressively decreased towards the 20% level and achieved stationarity after 144 h. In parallel, the percentage of cells in the G0 phase varied with a trend consistently opposite to that of S, trending to a final 70% value as in the control culture (Figure 1b). Finally, the percentage of cells in the G1 phase did not differ between C and I.

**Figure 2:**
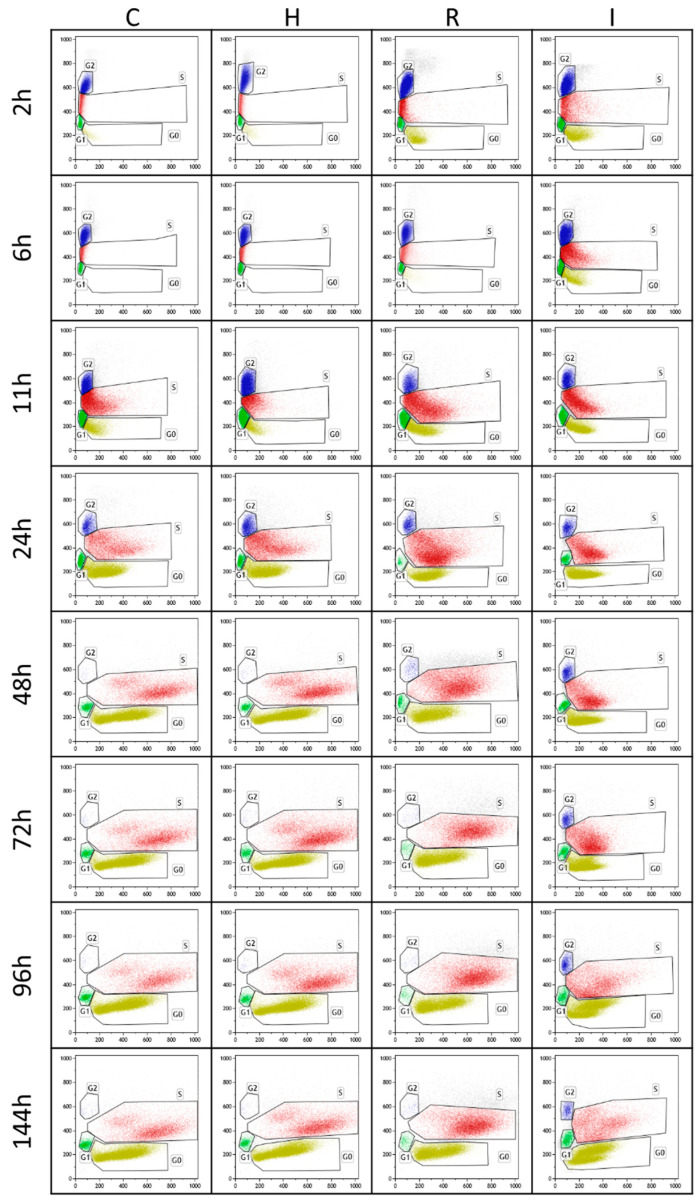
Bidimensional flow cytometric analysis at different times of the four growth conditions (C, H, R, and I). In the dot plots of each panel, G0, G1, S, and G2/M cell cycle stages are identified according to both forward scatter signal (FSC-A, *x*-axis) and green fluorescence (FL1-A, *y*-axis), representing cell size and DNA content per cell, respectively.

The bidimensional dot plots reporting the SYTOX fluorescence of the yeast cells versus the forward scatter of their size are reported during cultivation for each growth condition (C, H, R, and I) (Figure 2). It is clear from the sequential picture display that H and R treatments resemble the cell cycle of the control along the whole experiment. A slight difference between these treatments and the control is evident only at 2 h, with a higher presence of G0 cells than the control in the R treatment (similar, in this case, to the I treatment). Comparatively, the I treatment was dramatically delayed in the development of the different phases, with clear maintenance of a G2/M cell fraction until 144 h. It can also be noted that in C and H growth conditions, starting from 48 h, there was a split of the population of S phase cells into two differently sized subsets.

**Figure 3:**
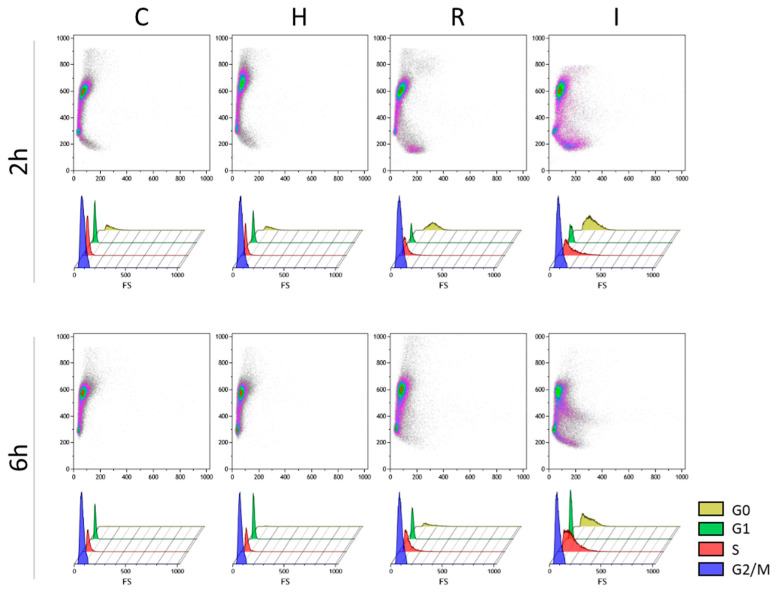
Bidimensional flow cytometric analysis of the early response of the four growth conditions (C, H, R, and I). In the three-dimensional dot plots of each panel, the green fluorescence (FL1-A, *x*-axis) and forward scatter signal (FSC-A, *y*-axis) results are presented, representing cell size and DNA content per cell, respectively. Relative amounts of cells in G0, G1, S, and G2/M cell cycle stages are depicted in the bottom rows for each time.

Focusing on the early hours of growth after the inoculum, Figure 3 shows a different 3D representation of the cytofluorimetric results at 2 and 6 h. In C, H, and R conditions, the start of cell proliferation corresponds to the depletion of G0 cells and an increase in G1, as well as a reduction in the S phase with an increase in the G2/M fractions of the cell population. Meanwhile, in the case of the I treatment, the fraction of G0 cells remains relatively high due to the lower duplication rate. At the same time, the number of cells in the S phase increases compared to the control and other treatments.

### 3.2. Transcriptomic Analysis of Early Response (0–24 h)

**Figure 4:**
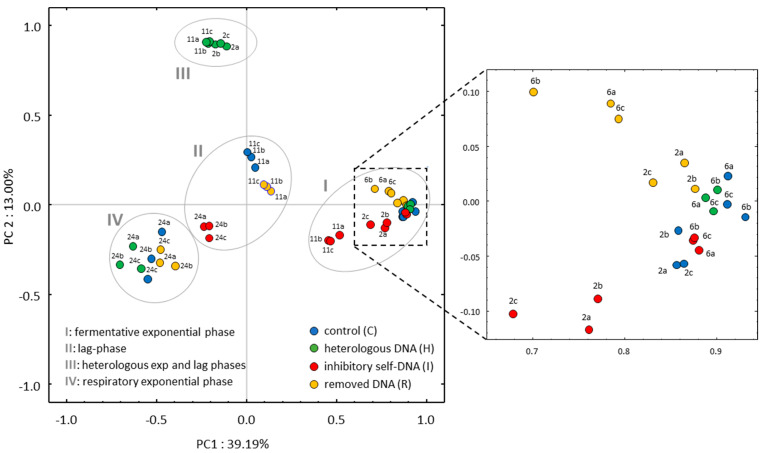
PCA ordination of transcriptomic data of *S. cerevisiae*. Each point represents a biological replicate of the four growth conditions of the batch cultures at different time points (2, 6, 11, and 24 h). Roman numerals indicate the resulting clusters, as reported in Figure S1.

The results presented in Figure 4 and Figure S1 demonstrate the consistency of the replicates within the different treatments. Moreover, four main treatment groups (I to IV) emerged along the first two Principal Components, which can be related to the growth phases and corresponding metabolic status (see Figure 1a). Group I contains the samples of the early fermentative phase for C, I, and R at both 2 and 6 h, whereas only the H treatment at 6 h is included. Group II comprises C and R samples at 11 h and I samples at 24 h, i.e., those samples corresponding to the lag phase of each treatment (see Figure 1a). Interestingly, group III is separated along the second component, aggregating the samples of the H treatment at 2 and 11 h. Finally, group IV puts together all samples at 24 h of the C, H, and R treatments, all corresponding to the beginning of the respiratory phase (see Figure 1a). In conclusion, the first PC axis, accounting for 39.2% of the total variance, clearly reflects the timing of the batch culture. Differently, the second PC axis, representing 13% of the total variance, is separating the heterologous treatment at two specific time points.

The number of Differentially Expressed Genes (DEGs) in the three treatments (H, I, and R) compared to the control (C) are reported in Table 1.

The number of DEGs in H (2356) at 2 h was more than one-third of the total number of yeast protein-coding genes (6600 as defined in the reference *S. cerevisiae* annotation, v. 3), with higher figures in the number of upregulated genes (1370) compared to the downregulated ones (986). Interestingly, at 6 h, only four upregulated genes were detected, while the number of DEGs increased again at 11 h (2278) with similar trends as at 2 h. No DEGs were found at 24 h in the H treatment. This corresponds to the reported multivariate analysis, clustering together the 2 and 11 h time points of the H treatment, separating them from the other clusters along the second PC axis (see Figure 4).

Comparatively, under the I growth condition, a much lower number of DEGs (130), in comparison to those found in the H treatment, was observed at 2 h, evenly distributed between up- and downregulated genes (67 and 63, respectively). The number of DEGs decreased at 6 h (72). Interestingly, however, the I treatment revealed the highest number of DEGs at this time point, when compared with the other two treatments. At 11 h, the total number of DEGs increased (829), becoming even higher at 24 h (1896).

Notably, in the R treatment we found no (at 2 and 11 h) or few (at 6 and 24 h) DEGs, indicating that, after the removal of exDNA, the exhausted medium did not substantially affect gene expression patterns compared to the control.

**Figure 5:**
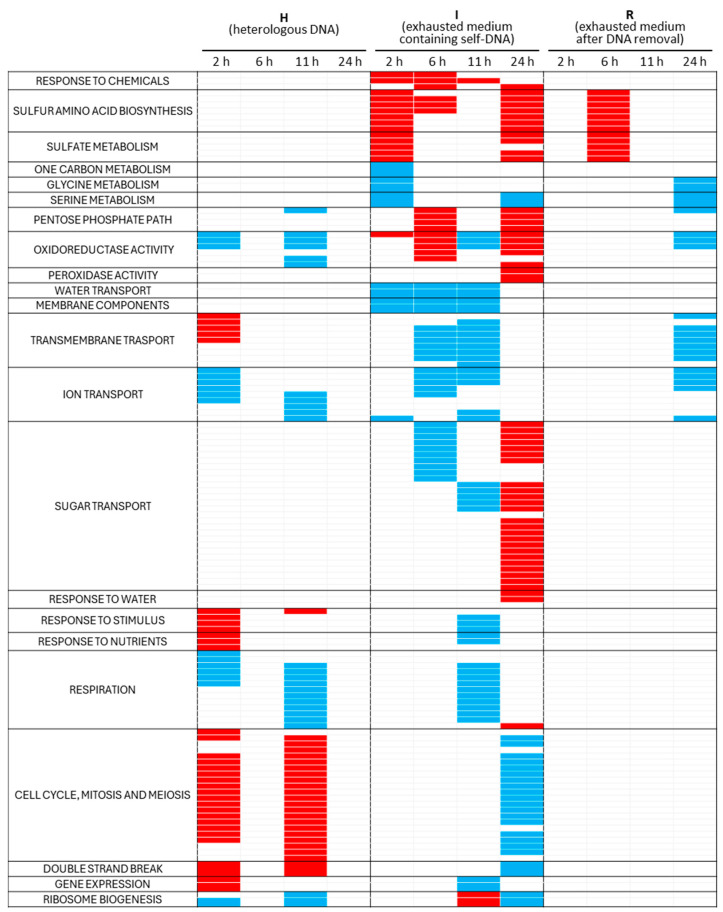
Summary of the GO enrichment analysis on filtered DEGs, with most enriched GOs (rows) grouped by functional process. The color of each cell in the columns (indicating treatment type and stage) shows the pattern of expression of the enriching genes (red: upregulated DEGs; blue: downregulated). In white, the absence of enrichment is shown.

The full description of gene ontologies enriched by up- or downregulated DEGs is reported in Table S1, whereas Figure 5 summarizes the main resulting trends.

In the I treatment, considering the GOs enriched by up- and downregulated DEGs per treatment per time point, respectively, a specific response to the chemical (cyclic organic compound/furfural) was associated with upregulated genes and revealed at 2 and 6 h. Enrichments from upregulated genes are also associated with sulfur amino acid biosynthesis and sulfate metabolism at 2 h, with sulfur amino acid biosynthesis GOs present at 6 h too, although reduced in numbers. The one-carbon, glycine, and serine metabolism GOs are associated with downregulated genes at 2 h. Interestingly, oxidoreductase activity is revealed to increase from 2 to 6 h, be downregulated at 11 h, and increase again at 24 h; in this last case, it is accompanied by the appearance of GOs associated with peroxidase activity. On the other hand, downregulated genes are associated with enriched GOs related to water transport and membrane components at 2, 6, and 11 h and with transmembrane, ion, and sugar transport mainly at 6 and 11 h. Interestingly, water response GOs appear to be enriched by upregulated DEGs at 24 h, together with upregulated DEGs associated with sugar transport.

The response to the H treatment appears to be rather different to that of the I one. Indeed, the response is reported as being associated with an external stimulus, response to nutrients, and in transmembrane and ion transport at 2 h. Respiration is enriched by downregulated genes at both 2 and 11 h, while an evident enrichment in cell-division-related ontologies (cell cycle, mitosis, and meiosis), as well as in double-strand break, is evident at 2 and 11 h. In particular, cell cycle and gene expression are mainly upregulated at 2 h.

The R treatment does not show any enrichment at 2 and 11 h, remaining similar to the control. Instead, at 6 h, upregulated DEGs determine an enrichment of sulfur amino acid biosynthesis and sulfate metabolism, similar to the I treatment at 2 h and 24 h. Furthermore, the R treatment presents some downregulation at 24 h in transmembrane and ion transport, glycine and serine metabolism, and oxidoreductase activity, while remaining identical to the control for all other GOs (Figure 5).

Table S2 includes all DEGs and their fold changes per treatment per time in the early response (2 to 24 h), while Table S3 reports the DEGs associated with main pathway cascades, as highlighted by the results.

Notably, of the 67 proteins upregulated at 2 h in the I treatment, 17 have uncharacterized or dubious functional assignments. Among the remaining, four genes (*YJR010W*; *YJR137C*; *YKL001C*; and *YLR303W*) are directly involved in sulfur metabolism and fifty-one have a conserved motif in their promoter associated with the transcription factory Yap-1. The yeast activator protein (Yap) family of b-ZIP proteins comprises eight members, with significant sequence similarity to the conventional yeast AP-1 protein. Among these, Yap4 was differentially expressed at 3 h in the I treatment. This protein is associated with pleiotropic drug resistance (PDR), is localized in the nucleus under oxidative stress, and is sequestered in the cytoplasm under reducing conditions mediated by NAD(P)H. Among the upregulated genes, other PDR genes are also present, plus genes involved in the uptake of cysteine, leucine, isoleucine, and valine. Of the 63 genes downregulated at 2 h, it is important to underline that 18 have uncharacterized or dubious functional assignments. There is a general downregulation of genes involved in one-carbon metabolism and in purine biosynthesis, accompanied by a general decrease in amino acid intake and transmembrane transport.

Considering the pathways reported in Table S3, it is evident that upregulated DEGs specific to the I treatment are associated with the response to weak acids and detoxification of furfural. This is also accompanied by the upregulation of *ADH6* (NADP dependent alcohol dehydrogenase) since 6 h, which remains upregulated in the early phase of the I treatment. Comparatively, *ADH4* and *ADH7* are upregulated at 2 h and 11 h in the H treatment. The stress-stimulated gene *GRE2*, encoding NADPH-dependent methylglyoxal reductase, is also specifically upregulated since the 2 h time point in I treatment, presumably enabling the conversion of furfural-like compounds.

Membrane ATPase may also contribute to the response to lower internal pH, removing protons outside the cell. The differential expression of pleiotropic drug resistance genes highlights that the only upregulated DEGs in the first two time points of the I treatment are *PDR5* and *PDR12*, while a wider pattern of up- and downregulated genes in this family is evident in the H treatment. Among the genes encoding the family of YAP transcription factors that are known to be involved in the PDR regulation, only *Yap4/Cin5*, generally associated with osmoregulation, is upregulated in I at 2 h, becoming upregulated also at 24 h.

The possible NADP+/NADPH imbalance due to acid compounds requires maintenance of oxidoreduction homeostasis and presumably also methionine and sulfur amino acids metabolism, accompanied by sulfur assimilation [32]. The sulphate assimilation and the sulfur amino acid metabolism GOs are enriched in the I treatment at 2 and 24 h and in R at 6 h (Figure 5). Among the genes associated with these GOs, we found an overlapping pattern between all treatments, although only I and the R enriched the corresponding GOs, with peculiar trends of DEGs in each treatment. In particular, *Mmp1* (S-methyl methionine permease) was not differentially expressed in I, while it was upregulated in both H and R. Differently, *Met32*, a zinc-finger, DNA-binding transcription factor associated with methionine biosynthesis regulation, was upregulated only in I.

The downregulated genes enriching the one-carbon metabolism GOs at 6 h in the I treatment are associated with serine and glycine catabolism. In particular, both *GCV1* and *GCV2*, glycine decarboxylases operating in mitochondria, show the lowest fold changes at this time point in the treatment, presumably to preserve glycine from degradation.

Interestingly, all the genes associated with the oxidative branch of the pentose phosphate pathway (PPP), that may contribute to the NADP+/NADPH balance, are all upregulated DEGs at 6 h and again at 24 h in the I treatment, while no DEGs result in the non-oxidative branch at the first three time points. NADPH produced by PPP is assumed to contribute to the reduction of sulfur bonds of glutathione during the oxidative stress response, although no DEGs are associated with glutathione metabolism in the very early stages of the I treatment. However, a pattern of DEGs is evident at 24 h, putatively caused by increasing oxidative stress, as shown by an increasing number of HSP genes at the same time point.

The upregulation of several different processes at 24 h in the I treatment highlights the dynamics of transcriptional and translational responses. This is associated with a significant upregulation of ribosomal proteins, both for nuclear and mitochondrial reservoirs, starting from nuclear encoded ones at 11 h. Transport of organic compounds, including sugar molecules, is also evident at 24 h.

Considering the response to the H treatment, the involvement of TOR genes is evident, with *TOR1* and *TOR2* upregulated since 2 h. In parallel, *Snf1*, *Snf3*, and *Snf5* are upregulated too, thus demonstrating the energy requirement in the very early phase of the treatment. Interestingly, the *TOR* genes and *Snf5* are upregulated in H at 11 h, when the effective diauxic shift from fermentation to respiration should have occurred, activating the glucose-repressed gene pattern and respiration.

Table S3 also shows the gene expression profiles associated with the major pathways of the yeast population exposed to the I treatment over the whole batch culture (from 2 to 144 h).

## 4. Discussion

The recently published research paper by [15] reported a metabolism-specific inhibitory effect of secreted self-DNA in fed-batch yeast cultures. This corresponded to a cessation of cell proliferation, despite nutrient availability, with a large proportion of the yeast population in the S phase of the cell cycle, as determined via cytofluorimetric analysis performed along the whole fed-batch cultivation run. In this study, we performed in-depth cytofluorimetric and transcriptomic analyses of the yeast *S. cerevisiae* in a batch experiment, exposed to the exhausted medium of the above-mentioned fed-batch culture containing self-DNA secreted fragments. Indeed, close analysis of the growth curve of a microbial species has historically been used an essential tool in the study of cell physiology: “the growth of bacterial cultures, despite the immense complexity of the phenomena to which it testifies, generally obeys relatively simple laws, which make it possible to define certain quantitative characteristics of the growth cycle” [33].

Therefore, on one hand, a very significant decrease in the specific growth rate of the yeast population was observed during the fermentative phase, providing further evidence of the inhibitory effect of the medium containing the yeast secreted DNA fragments (I treatment). On the other hand, the removal of such exDNA from the growth medium (R treatment) completely canceled the inhibitory effect, resulting in a growth curve for this treatment that was almost identical to the control (C). Also, the exposure to high concentrations of heterologous DNA did not produce any detectable effect on the growth curve, again producing results similar to the control. This last result, while confirming the findings reported by [15], again demonstrates a lack of growth inhibition by extracellular nonself-DNA.

Interestingly, in the culture exposed to the inhibitory self-DNA, after a very long diauxic lag phase (35 h), apparently due to the prolonged inhibitory effect established in the fermentative exponential phase, a resumption of growth was observed. The second exponential growth phase, based on the switch to a respiratory metabolism on ethanol, reached the final plateau of biomass after 144 h, much later compared to the 50 h time point of the control and the other treatments.

At the completion of the fermentative phase, the I treatment reached a significantly lower biomass compared to the control and the other treatments (see beginning of the lag phases in Figure 1a), which evidently corresponds to a loss of efficiency of sugar conversion to biomass. It is known that, in normal aerobic growth conditions, respiration contributes to mass production for about 3–20% of carbon source utilization, including during the fermentative phase [34]. Then, the lower observed biomass plateau could be indeed be explained by an impairment of mitochondrial functionality caused by the oxidative stress induced by self-DNA (see enrichment of oxidoreductase GOs in Figure 5).

Regarding the elongated diauxic lag phase in the inhibited culture, it is known that in yeast the efficiency of the activation of mitochondria, particularly of the complexes III and IV of the electron transport chain, represents the major bottleneck for lag phase duration [35,36]. Thus, the abnormal elongation in the I culture, as well as a relatively slower growth rate in the subsequent respiratory phase, suggests a carry-over of impairment of mitochondria functionality, likely due to ROS enrichment in the inhibited cells. It is possible to speculate that another factor that could affect the longer length of the diauxic phase might be the longer time of exposure to a repressive glucose concentration [36]; this occurred in the I culture because of the slower glucose consumption during the inhibited fermentative phase.

Cytofluorimetric analysis of the cell cycle clearly showed that the control culture (C), as well as the other two cultures exposed to either heterologous DNA (H) or to supernatant after self-DNA removal (R), rapidly activated cell division after the inoculum. This is shown by the higher percentage of cells in the G2/M phase of the control compared to the I treatment during glucose fermentation and up to the lag phase (Figure 1b,c). Then, these cultures shifted their metabolism to respiratory growth on ethanol, achieving stationarity with a complete cessation of cell mitosis and final values of G0 and S phases above 70% and 20%, respectively, at the final growth plateau, when all the available nutrients were consumed from the medium. Comparatively, in the case of the yeast cultured in the presence of self-DNA, the cells were strongly inhibited in the early division process, with the G2/M fraction largely reduced in the population. During the fermentative phase, the fraction of cells in the S phase reached higher values than the control, but then they progressively decreased towards the 20% level and achieved stationarity after delayed respiratory recovery, confirming the effect of self-DNA inhibition on the cell cycle. This is in agreement with other experimental observations of the arrest of growing yeast populations during the S phase [15,37,38,39], likely involving the intra S phase check point due to stalling replication and impairment of normal replisome processivity [40,41].

Considering the transcriptomic analysis, the early response to the presence of extracellular DNA in the growth medium corresponded to peculiar patterns of gene expression for both the self- and (heterologous) nonself-DNA exposure treatments. The early response to the different treatments revealed a different number of DEGs per treatment per time. Specifically, the highest number of DEGs occurred in the H treatment at 3 and 11 h, when compared to the I treatment, while the R treatment showed no DEGS at these two time points, revealing its behavior as being comparable to the control in the early growth stages. Interestingly, however, the R treatment showed a small number of DEGs at 6h. Worthy of note is the remarkable relative number of uncharacterized/dubious function assignments of the genes involved in the response.

GO enrichment analysis revealed that sulphate metabolism and sulfur amino acid biosynthesis are upregulated by DEGs in the R treatment at 6 h and in the I treatment at 2 h. The DEGs associated with these GOs, showed an overlapping pattern among some of the genes in all treatments. Focusing on the non-overlapping genes, a peculiar trend per treatment can be highlighted. In particular, the upregulation of Mmp1 (S-methyl methionine permease) only in H and R shows different dynamics in sulfur assimilation compared to the reduction of this process in the I treatment. On the other hand, only in I, we observed the upregulation of Met32, a zinc-finger, DNA-binding transcription factor associated with methionine biosynthesis regulation and a notorious actor in cell cycle arrest [42]. This supports the evidence that the one-carbon metabolism GOs also appear to be associated with downregulated genes, thus preventing the loss of methionine and the reductive power exerted by sulfur amino acids, which is required for the NADP+/NADPH balance. It is well known that one-carbon metabolism regulates methionine cycles and folate integrates glucose, amino acids, and vitamins [31]. In addition, it also supports the synthesis of relevant macromolecules like nucleotides, lipids, and proteins, the substrates for methylation reactions, and the maintenance of redox balance [31,43]. The carbon units that fuel this specific pathway may be obtained from specific amino acids, such as serine, glycine, and threonine, or can be synthesized *de novo* from glucose, exploiting the first two steps of glycolysis towards the serine synthesis pathway [31,43,44] in the presence of sulfur. However, the overall picture highlighted in I resembles a typical methionine requirement that could be associated with lower sulfate assimilation and also with a reduced transmembrane transport process, which appears to be solved at the 24 h time point, when positive regulation of carbohydrate transport appears to be evident.

Regarding the cell-cycle-related GOs, there are no evident changes during the first exponential growth period in the I treatment. This may seem counterintuitive considering the significantly reduced growth rate of the culture in this condition. The explanation for this phenomenon can be found in the higher expression of sulfur amino acid biosynthesis and sulphate metabolism with the MET32 gene, a notorious actor of cell cycle arrest, being strongly upregulated. On the other hand, the significant downregulation of glycine, serine, and one-carbon metabolism GOs reflects the lower amino acid availability as well as the reduced nucleotide metabolism associated with the lower rate of cell duplication (Table S1).

It is interesting to note that self-DNA removal from the medium (R treatment) mostly recreates the conditions of control via the absence of most DEGs observed in the I treatment, thus constituting an indirect demonstration of the distinctive inhibitory effects of extracellular self-DNA on cell functionality mechanisms.

Regarding the transcriptomic results in the case of the H treatment, there are very clear responses to the stimulus and nutrients, as well as an impact on transmembrane transport activation. Furthermore, the downregulation of nucleotide metabolism can be interpreted as a negative feedback on these production pathways, due to the available nucleotides derived from the uptake of heterologous DNA, which represent a valuable source of functional building blocks. In this situation, yeast cells show a generalized upregulation of gene expression associated with cell division and cell cycle processes. Despite the above mentioned “biostimulation” by nonself-DNA and the activation of extensive transcriptional dynamics, the emergent property of the growth rate did not differ from the control, which was already at the maximum level for the used yeast strain. In summary, the reaction to extracellular heterologous DNA corresponded to the activation of many cell pathways that did not cause an inhibitory effect on cell growth, which is noteworthy considering the high concentration applied. The general downregulation of genes associated with transport at both 6 and 11 h in the I treatment followed by upregulation at 24 h, which was reactivated during the long diauxic lag phase, agrees with what was observed in other experiments on self-DNA inhibition in different model organisms. Indeed, in the case of the plant model of *Arabidopsis thaliana,* a drastic reduction in membrane permeability was observed, associated with a calcium spike at the membrane level, as well as a response to ROS mainly associated with organelles misfunctioning (both chloroplasts and mitochondria), putatively due to an inhibition of the retrograde signaling from organelles to the nucleus [45,46,47]. Moreover, in the same plant model, metabolomic studies demonstrated that self-DNA inhibition induced a generalized suppression of transcription processes and caused an accumulation in the cells of RNA constituents, along with AMP and GMP, with their cyclic analogues and methylated forms [48]. Notably, in the present work, the recovery from inhibition was found to be associated with energy requirements corresponding to an increased cAMP content in the cell.

Different studies on other model organisms exposed to both extracellular self- and nonself-DNA revealed specific phenotypic effects, which can be considered comparable with the observed cell cycle impairment in this work on yeast. Indeed, in *Drosophila melanogaster*, self-DNA induced a strong reduction in the fertility of adult insects, associated with an enhanced production of the metabolite pidolate, a compound known for its effects on altering meiosis during egg formation [49]. Moreover, in the model nematode *Caenorhabditis elegans*, the inclusion of self-DNA in the diet of adult worms again resulted in altered egg deposition, with enhanced production of RAD-51, a protein involved in DNA damage repair, in the gonad meiotic process [50].

Another highly remarkable result of the transcriptomic analysis is associated with the gene expression profile and DEGs associated with endocytosis and membrane transport processes. In fact, during the first exponential phase characterized by fermentative metabolism, it is evident that exposure to heterologous DNA corresponded to a significant activation of genes involved in these processes, as well as to corresponding ontologies showing the active handling of nonself-DNA. On the other hand, exposure to self-DNA appeared to downregulate most of these molecular pathways, with the exception of a few genes that are hexose transporters (HXT1) and membrane proteins associated with pleiotropic drug resistance (PDR12 and PDR5)**.** Worthy of note is that the same upregulation of PDR12 was highlighted in *A. thaliana* seedlings exposed to self-DNA [45].

Interestingly, activation of the repressed endocytosis genes during fermentation becomes evident in the self-DNA treatment (I) only during the second exponential phase after switching to the respiratory metabolism (see Figure 1a). This is remarkable and may be explained as the capability of the cells to recover from inhibition by extracellular self-DNA fermentative fragments [15], only when the switch to the respiratory metabolism has been completed, after the diauxic lag phase. In other words, we suggest that the secreted fermentative DNA fragments, because of their specific inhibitory effect, could block the absorption of cells that show the same fermentative metabolism. But, once these same cells switch to a different metabolism, i.e., switching to respiration, they become able to process such inhibitory exDNA fragments.

Following the same reasoning, this result also provides a putative solution for an unexplained observation published by [15]. In fact, that study reported the accumulation of ssDNA fragments in the medium, mapping onto active genome regions in the metabolism displayed by the yeast cells. Such specific fragments collected in the early hours of the respiratory phase of the fed-batch run were not present in the supernatant samples collected during the subsequent fermentation phase. This can be logically explained by the cell uptake, only after switching to fermentative metabolism, of the previously secreted fragments during a different active metabolism. In other words, self-DNA fragments are recognized as inhibitory, and consequently not absorbed, by cells expressing the corresponding genomic regions (i.e., the same metabolism). Comparatively, after switching to a different metabolism, the same cells no longer recognize those ex-DNA fragments as self-DNA and so become able to process them via active endocytosis, as always performed in presence of heterologous DNA.

In the case of the fed-batch experiment by [15], this was observed during the switch between respiratory and fermentative metabolisms. In this study, the opposite switch was analyzed, with a change from fermentative metabolism to respiratory functional metabolism, still showing a consistent change in the uptake and metabolization of exDNA.

## 5. Conclusions

This work sheds light on the inhibitory effect of self-DNA on the model organism *S. cerevisiae* as a dynamic process, with initial impairment of growth and cell cycle, followed by recovery of the functional activity. These findings expand the current understanding on the generality of the extracellular self-DNA inhibitory effect on cell proliferation and represent the basis for further investigations on yeast as well as on other organisms for both basic science and biotechnological applications.

## Figures and Tables

**Table 1:** Total number of DEGs per treatment and time point, as well as up- and downregulated genes.

		DEGs			
		2 h	6 h	11 h	24 h
H	TOTAL	2356	4	2278	0
	up	1370	4	1186	0
	down	986	0	1092	0
I	TOTAL	130	72	829	1896
	up	67	53	289	911
	down	63	19	540	985
R	TOTAL	0	18	0	134
	up	0	16	0	43
	down	0	2	0	91

## Data Availability

The original RNA-seq datasets used in the study are publicly available at NCBI and accessible at BioProject Id PRJNA1111483. Other original contributions presented in the study are included in the article/Supplementary Materials. Further inquiries can be directed to the corresponding author/s.

## Supplementary Materials

The following supporting information can be downloaded at: https://www.mdpi.com/article/10.3390/biom14060663/s1, Figure S1: Numerical clustering of transcriptomic data of *S. cerevisiae*. Each point represents a biological replicate of the four growth conditions of the batch cultures at different time points (2, 6, 11, and 24 h). Grey Roman numerals indicate the main functional groups (see also Figure 4); Table S1: GO enrichment analysis on filtered DEGs, with most enriched GOs (rows) grouped by functional process; Table S2: DEGs per treatment per time point; Table S3: DEGs and gene expression per pathway.
