# Supplementary material for: Extracellular Self-DNA Effects on Yeast Cell Cycle and Transcriptome during Batch Growth
Source: Biomolecules. 2024 Jun 6;14(6):663. doi: 10.3390/biom14060663 (PMC11201494; doi:10.3390/biom14060663)
Supplement: Supplementary file 1 [file biomolecules-14-00663-s001.zip › Figure S1.pptx]

## Slide 1
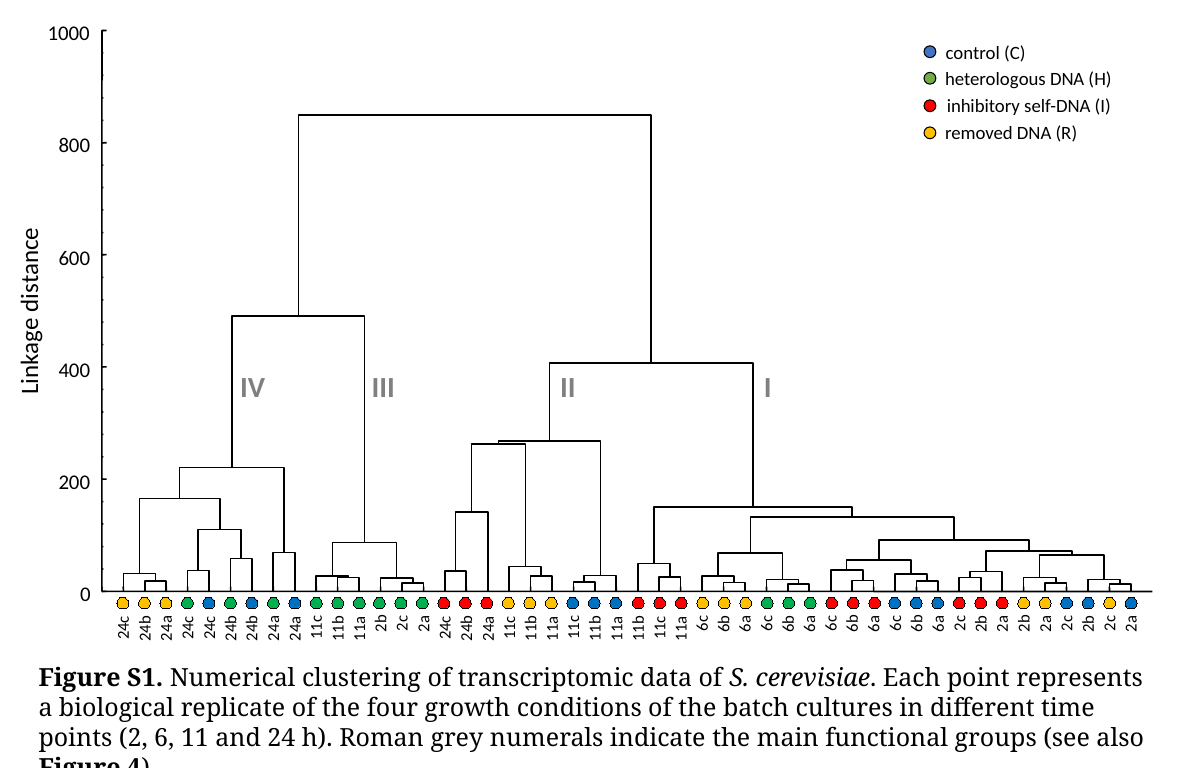

1000
control (C)
heterologous DNA (H)
inhibitory self-DNA (I)
removed DNA (R)
800
600
Linkage distance
400
IV
III
II
I
200
0
2c
6c
6c
6c
6c
2c
2c
2c
2b
2a
6b
6a
6b
6a
6b
6a
6b
6a
2b
2a
2b
2a
2b
2a
24c
24c
24c
11c
24c
11c
11c
11c
24b
24a
24b
24b
24a
24a
11b
11a
24b
24a
11b
11a
11b
11a
11b
11a
Figure S1. Numerical clustering of transcriptomic data of S. cerevisiae. Each point represents a biological replicate of the four growth conditions of the batch cultures in different time points (2, 6, 11 and 24 h). Roman grey numerals indicate the main functional groups (see also Figure 4).
